# Spatial-Temporal Feature Analysis on Single-Trial Event Related Potential for Rapid Face Identification

**DOI:** 10.3389/fncom.2017.00106

**Published:** 2017-11-27

**Authors:** Lei Jiang, Yun Wang, Bangyu Cai, Yueming Wang, Yiwen Wang

**Affiliations:** ^1^Qiushi Academy for Advanced Studies, Zhejiang University, Hangzhou, China; ^2^Department of Computer Science and Technology, Zhejiang University, Hangzhou, China; ^3^Department of Biomedical Engineering, Zhejiang University, Hangzhou, China; ^4^Department of Electronic and Computer Engineering, Department of Chemical and Biology Engineering, Hong Kong University of Science and Technology, Kowloon, Hong Kong

**Keywords:** event related potential, single-trial, spatial-temporal feature, brain-computer interface, rapid face identification

## Abstract

The event-related potential (ERP) is the brain response measured in electroencephalography (EEG), which reflects the process of human cognitive activity. ERP has been introduced into brain computer interfaces (BCIs) to communicate the computer with the subject's intention. Due to the low signal-to-noise ratio of EEG, most ERP studies are based on grand-averaging over many trials. Recently single-trial ERP detection attracts more attention, which enables real time processing tasks as rapid face identification. All the targets needed to be retrieved may appear only once, and there is no knowledge of target label for averaging. More interestingly, how the features contribute temporally *and* spatially to single-trial ERP detection has not been fully investigated. In this paper, we propose to implement a local-learning-based (LLB) feature extraction method to investigate the importance of spatial-temporal components of ERP in a task of rapid face identification using single-trial detection. Comparing to previous methods, LLB method preserves the *nonlinear* structure of EEG signal distribution, and analyze the importance of *original* spatial-temporal components via optimization in feature space. As a data-driven methods, the weighting of the spatial-temporal component does not depend on the ERP detection method. The importance weights are optimized by making the targets more different from non-targets in feature space, and regularization penalty is introduced in optimization for sparse weights. This spatial-temporal feature extraction method is evaluated on the EEG data of 15 participants in performing a face identification task using rapid serial visual presentation paradigm. Comparing with other methods, the proposed spatial-temporal analysis method uses sparser (only 10% of the total) features, and could achieve comparable performance (98%) of single-trial ERP detection as the whole features across different detection methods. The interesting finding is that the N250 is the earliest temporal component that contributes to single-trial ERP detection in face identification. And the importance of N250 components is more laterally distributed toward the left hemisphere. We show that using only the left N250 component over-performs the right N250 in the face identification task using single-trial ERP detection. The finding is also important in building a fast and efficient (fewer electrodes) BCI system for rapid face identification.

## Introduction

An event-related potential (ERP) is the brain response measured in electroencephalography (EEG) signal, which is evoked by a specific sensory, cognitive, or motor event (Luck, 2014). One of the important ERP components, P3, is elicited when people get involved in the process of target detection in the oddball paradigm (Polich, 2007), in which low-probability target items are mixed with high-probability non-target (or “standard”) items. The detection of P3 has been introduced to the area of brain-computer interface (BCI) that conveys subject's intention in real-time for different tasks, such as character spelling (Donchin et al., 2000; Sellers et al., 2006; Belitski et al., 2011) and direction control in wheelchair (Wang et al., 2005; Piccione et al., 2008; Li et al., 2010). Among those BCI studies, one of the interesting application is face identification (Cai et al., 2013). Face is particularly special among all images due to the development of primates' central neural system in recognizing and socializing with the same species (Gazzaniga et al., 2009). A face conveys large amount of essential information in social interaction, including identity, gender, age, emotional expressions. (Haxby et al., 2002). The automatic machine face identification has achieved good performance in the controlled environments with constrained illumination, poses, or facial expressions (Gao and Qi, 2005; Zhang and Gao, 2009; Wu et al., 2010; Chen et al., 2013), but is still far from satisfactory due to the non-rigid structure of face and the high degree of variability in the above conditions (Zhao et al., 2003; Tolba et al., 2006). In comparison, human has powerful ability in identifying faces. We can identify faces related to past experience using long term memory (Gosling and Eimer, 2011; Zheng et al., 2012). What is extraordinary is that our brain could perform face identification within a surprisingly short time duration with high accuracy across large viewpoint changes, under poor lighting conditions, even only partial views are available (Eger et al., 2005; Gosling and Eimer, 2011).

The face processing in human brain can be recognized by analyzing ERP in EEG signals. Due to the low signal-to-noise ratio (SNR) of EEG, the ERP studies in neuroscience or brain computer interface application are mainly based on grand-averaging (Luck, 2014), i.e., EEG signals in multiple trials with the same labels are averaged to reduce the noise among trials. The statistical test is generally performed to see if there is significant difference of the mean amplitudes on each temporal component between conditions. The grand-averaging approach may not be appropriate for the applications where there is no label information for averaging. In addition, the grand-averaging greatly reduces the efficiency of real-time implementation, and increases the fatigue of the subjects (Hoffmann et al., 2008; Krusienski et al., 2008). Therefore, single trial detection of the ERP is necessary for real-time BCI applications. Cai et al. proposed a BCI application to implement face identification based on single-trial ERP detection using face-related components, and demonstrated robust real-time performance in rapid serial visual presentation (RSVP) paradigm (Cai et al., 2013). Furthermore, a closed-loop BCI system was proposed for face retrieval in database by integrating the remarkable human recognition function with the fast searching ability by computer vision (Wang et al., 2015).

ERP demonstrates different features distribution temporally and spatially. Previous research has identified the temporal components that are closely related to face processing, such as the N170 component (Zheng et al., 2012), vertex positive potential (VPP) (Kai et al., 1995) and the late component FP600 (Eimer, 2000). Due to the fact that the N170 is insensitive to facial identity and familiarity, it is assumed that the N170 reflects the stages of structural encoding of faces prior to face identification (Rossion et al., 1999; Bentin and Deouell, 2000; Eimer, 2000; Gosling and Eimer, 2011; Zheng et al., 2012). To further investigate the ERP components associated with face identification, some studies have focused on the subsequent N250. In Gosling and Eimer's study (Gosling and Eimer, 2011), in contrast to the N170, the N250 elicited by famous faces was significantly larger than the N250 elicited by non-famous faces in an explicit face identification task. Similarly, in a experiment conducted by Zheng et al. (2012), participants performed a face identification task while face identity strength was manipulated by varying the relative weight of an individual face vs. the average face. This work reports that the increase of the face identity strength is associated with the larger N250, but the effect is absent in the N170. These studies all suggest that the N250 is an early ERP component associated with face identification task. In addition, other studies also report that, relative to unfamiliar faces, familiar faces elicit distinct FN400, an enhanced negativity over mid-frontal areas, which is believed to reflect the later activation of semantic or episodic memory (Curran and Hancock, 2007; Jon et al., 2011). On the other hand, using noninvasive neuroimaging techniques (Han et al., 2013, 2015a,b), such as functional magnetic resonance imaging (fMRI) and positron emission tomography (PET), a face-related brain region has been identified, the fusiform gyrus (Kanwisher et al., 1997; Mccarthy et al., 1997; George et al., 1999; Kuskowski and Pardo, 1999; Rossion et al., 2000; Loffler et al., 2005), when a face is shown among images with non-face topics such as object or national scene. Research also suggests that different hemispheres of brain could play roles non-symmetrically in identifying faces (Keenan et al., 2001). Therefore, understanding the spatial and temporal features of ERP could contribute to a fast and efficient BCI design.

Investigation on *both* spatial and temporal features of the ERPs may provide potentials using partially EEG electrodes and early temporal components to generate an efficient face identification application using single-trial ERP detection. It may also shed light on understanding the mechanism of robust face identification in brain. The classic grand averaging approach identifies the component where the temporal amplitudes are statistically significantly different between conditions (Gosling and Eimer, 2011; Zheng et al., 2012). Researchers also develop a few computational methods to analyze the spatial-temporal features of ERPs. The most common used methods are Fisher criterion (FC) (Krusienski et al., 2008; D'croz-Baron et al., 2012; Guo et al., 2015), support vector machine recursive feature elimination (SVMRFE) (Guyon et al., 2002; Hidalgo-Muñoz et al., 2013), and mutual information (MI) (Shahriari and Erfanian, 2011; Ang et al., 2012). FC evaluates the feature importance by linearly computing the ratio of inter-class scatter to the intra-class scatter for each feature. The bigger the ratio is, the more important the feature is. Krusienski et al. used FC to analyze important features for P300 speller BCI, and found the P300 features in the parietal electrodes contributed the most to the ERP detection (Krusienski et al., 2008). SVMRFE utilizes the weights of features in trained SVM model to indicate the feature importance. Hidalgo-Muñoz and his colleagues proposed to use SVMRFE to analyze the ERPs induced by visual stimuli categorized with different value of affective valence. They demonstrated that ERP features from 300 to 500 ms in the central, parietal and occipital electrodes were the most relevant for classifying the affective valence (Hidalgo-Muñoz et al., 2013, 2014). Liu et al. also adopted SVMRFE to analyze the ERP features in the emotion recognition task (Liu et al., 2014). The mutual information method is based on statistical theory, it measures the feature importance by evaluating the information amount between each feature and the class label. Shahriari et al. proposed to use mutual information to analyze important electrodes in P300 speller BCI, and demonstrated that using features in 8 important electrodes located in central areas can achieve comparable performance as the full electrodes (Shahriari and Erfanian, 2011). Zhang et al. used mutual information to analyze EEG features for mental fatigue analysis, and demonstrated alpha band power in the parietal and occipital electrodes contributed most to the fatigue level classification (Zhang et al., 2016). However, FC and SVMRFE have linear assumption, which cannot capture the nonlinear structure of the EEG signal, and the extracted features depend on the ERP detection models. Mutual information is data-driven and free of modeling, but the extracted features may not be sparse. In addition, all the above methods for feature extraction have not been fully validated in the single trial ERP detection application.

We are interested to investigate the early temporal components and the hemisphere distributional features of the ERPs in order to design an efficient face identification task. In this paper, we propose to implement a local-learning-based (LLB) feature extraction method (Sun et al., 2010) to investigate the importance of spatial-temporal ERP features in the face identification task. Fifteen participants were instructed to perform a face identification task when face images were presented through the rapid serial visual presentation (RSVP) paradigm. The face images of 36 celebrities varied in illumination, poses and expressions, even were partially occluded. The corresponding EEG signals were simultaneously collected and analyzed by the proposed spatial-temporal feature extraction method. The LLB method has been used in gene selection for cancer classification (Sun et al., 2010). In this paper, the LLB method decomposes the nonlinear EEG signal into a feature space by weighting the local structure of single trial ERP features, where the weights indicate the importance of the features over spatial distribution and temporal course. The weights are obtained by minimizing the distances within the face images of the same identity and maximizing distances from the face images of other identities. With the weight decay operation, the extracted spatial and temporal features are constrained to be sparse. The temporal-spatial importance distribution of the ERP components are also compared with FC, SVMRFE and mutual information (MI). The contributions of the important features are validated by applying the linear discriminant analysis (LDA) (Bishop, 2006), linear SVM (linear-SVM), and kernel SVM (K-SVM) (Rakotomamonjy and Guigue, 2008) on the single-trial ERP detection using only the extracted spatial-temporal components. The area under the receiver operating characteristic (ROC) curve (AUC) is used as the target identification performance criterion (Fawcett, 2004) considering the false positive rate (FPR) and the true positive rate (TPR). More specially, we investigate the earliest ERP component over temporal course and the spatial distribution over different hemispheres with respect to the rapid face identification.

The paper is organized as follows: in section Methods, face identification task and EEG data collection are described, followed by the descriptions of the spatial-temporal feature extraction by the LLB method, target/non-target face classification by single-trial ERP detection and performance evaluation criterion. Our results are reported in detail in section Results. Discussion and conclusion are in section Conclusion and Discussion.

## Methods

### Face identification and experiment procedure

**Figure 1:**
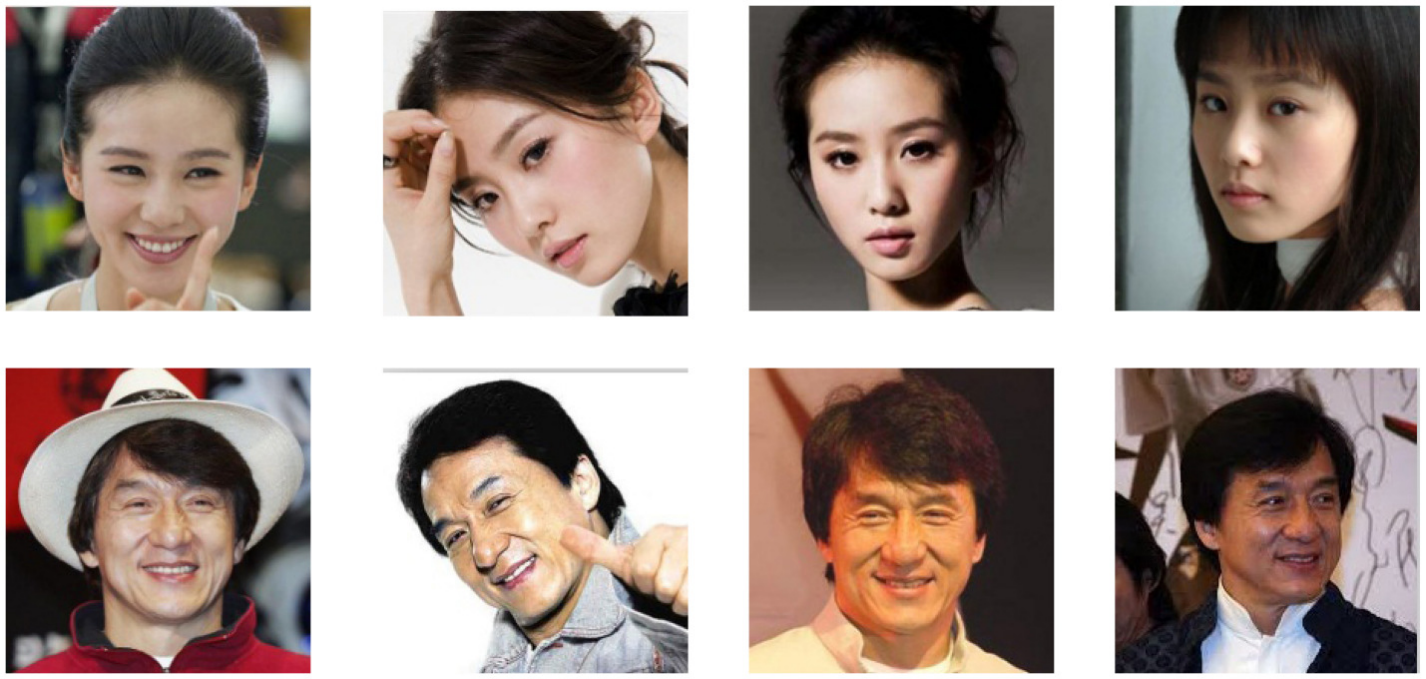
Examples of the face images used in our experiment. Each row shows 4 different exemplars of a celebrity.

Face images used in the identification task were selected from Google Images, and consisted of 540 different color images of 36 celebrities (18 females, 18 males), 15 different exemplars for each celebrity. The ratio of the familiarity vs. unfamiliarity of the celebrities was 47.82 vs. 52.18% in average across participants. In order to exclude non-facial cues of the identity of each image, all face images were cropped and scaled to 200 × 200 pixels using Adobe Photoshop. The face images varied in illumination, poses and facial expressions, even were partially occluded (Cai et al., 2013). The examples of the face images used in our experiment are illustrated in Figure 1.

Totally 15 participants (7 males, 8 females, aged 21–25) participated in this study. The experiments were approved by the ethics committee of Zhejiang University. The experimental setup complied with generally accepted guidelines for ERP study documented in (Picton et al., 2000). During the experiment, participants were seated comfortably in a dark and quiet cabin, about 90 cm in front of a computer screen. The whole experiment consisted of 12 blocks of target face identification task. In each block, one celebrity was randomly selected from 36 candidate celebrities, and served as target needed to be identified by the participants. Each block consisted of 160 face images, including 10 face images of the chosen target and 150 face images that were randomly selected from the rest 35 celebrities. All the 160 face images were presented in a random order to the participants in the rapid serial visual presentation paradigm (Cai et al., 2013), each face image was presented for 500 ms, followed by a blank inter-stimulus interval (ISI) of 500 ms. Participants were required to minimize body and eye movements, maintain central eye fixation, and press left button as quickly and accurately as possible when they identified the target faces. Breaks were encouraged between blocks. The whole experiment lasted about 2.5 h.

### Data recording and analysis

#### Data recording and preprocessing

**Figure 2:**
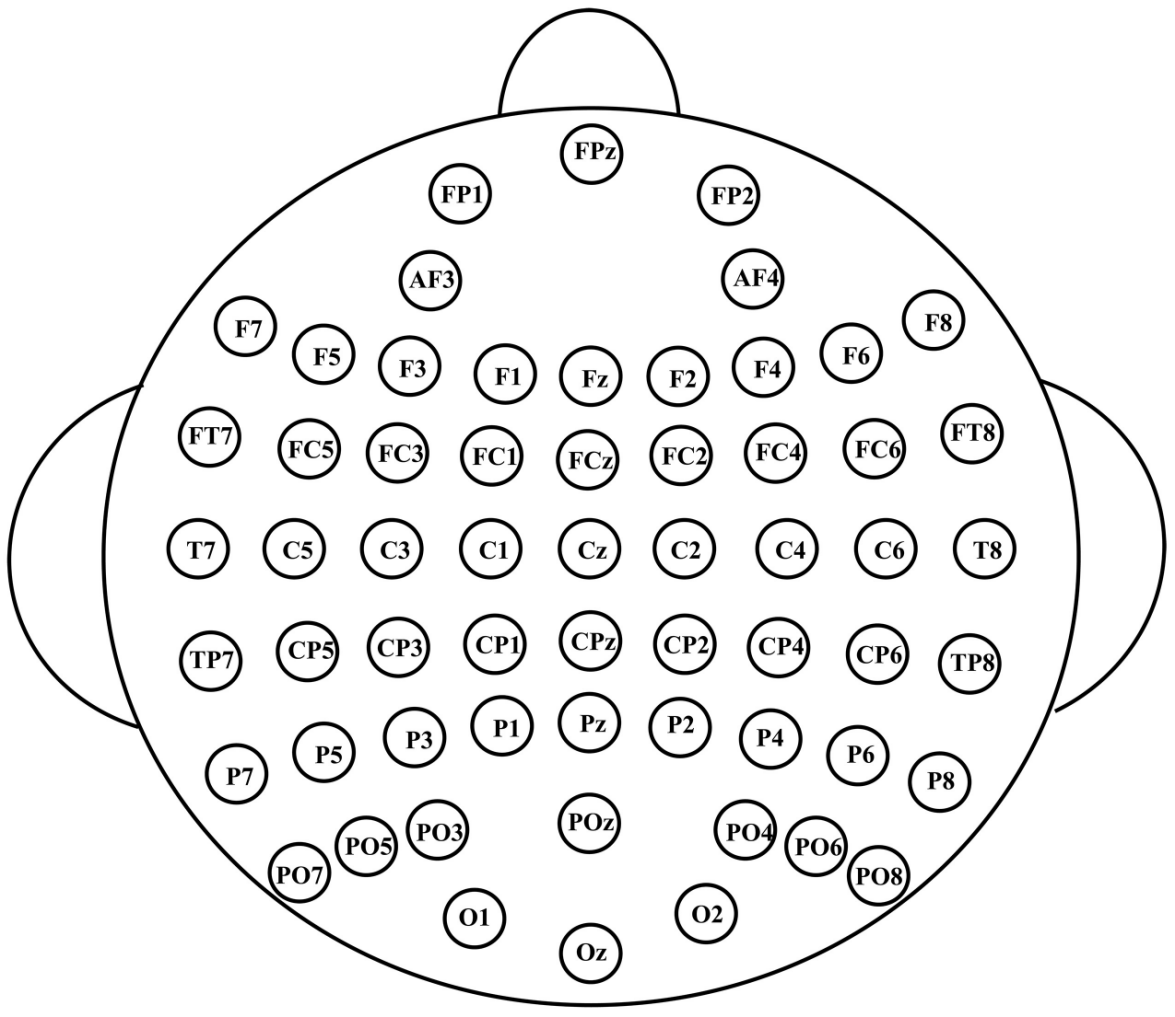
The scalp distribution of the electrodes used in our experiment.

The EEG signals were recorded with 60 Ag/AgCl electrodes using NeuroscanSynamps system. Figure 2 shows the scalp distribution of the 60 electrodes, electrode positions included the standard 10–20 system locations and intermediate positions (Luck, 2014). EEG signals were recorded at the sampling rate of 1,000 Hz, with a 200 Hz low-pass filter and a 50 Hz notch filter. EEG signals were referenced to the nose, Afz served as ground. Impedances were kept below 30 kΩ. Blinks were monitored by vertical electrooculogram (EOG) electrodes located above and below the left eye. EEG signals were further pre-processed to remove EOG artifacts using the correlation between EEG and EOG (Chen et al., 2010). A Butterworth band-pass filter was used to filter the EEG signals between 0.5 and 30 Hz. Then EEG signals were segmented into epochs from 0 to 800 ms after stimuli onset, and the baseline was corrected relative to the 200 ms pre-stimulus interval.

For spatial-temporal feature extraction and target face identification, EEG epochs were downsampled from 1,000 to 100 Hz (i.e., 80 samples in an 800-ms EEG epoch). Then the EEG epochs were concatenated by channel for each face stimulus, creating a feature vector which represented the EEG spatial-temporal pattern corresponding to each face stimulus. The dimension of the vector was 4,800 (60 channels × 80 samples).

#### Local-learning-based spatial-temporal feature extraction

In this session, we describe in detail how to adopt a LLB feature extraction method (Sun et al., 2010) to extract important spatial-temporal features that contribute to single-trial ERP analysis.

Let $D={\left\{\left({\text{x}}_{n},y_{n}\right)\right\}}_{n=1}^{N}\subset {\mathbb{R}}^{K}\times \left\{\pm 1\right\}\;$ be the training sample set of a certain participant's EEG signals, where *N* is the number of the training samples, *n* is the sample index, **x**_*n*_ is a *K* dimensional EEG original signal vector (*K* = 4800 in this paper), *y*_*n*_ is its corresponding target/non-target class label. We define two patterns *NH*(**x**_*n*_) and *NM*(**x**_*n*_). *NH*(**x**_*n*_) is the most similar pattern to **x**_*n*_ with the same class, and *NM*(**x**_*n*_) is the most similar pattern to **x**_*n*_ with the other class label. Here the similarity is measured by Manhattan distance (Sun et al., 2010). The idea is to assign a weight to each feature to represent its importance, so as to maximize inter-class distance and minimize intra-class distance in the feature space. For the sample **x**_*n*_, we define *L*(**x**_*n*_|**w**) as the difference between the intra-class distance and inter-class distance when weighting the importance of each EEG spatial-temporal feature by nonnegative row vector **w**^**2**^:

(1) $$L\left(x_{n}|w\right)=w^{2}\left|x_{n}-NM\left(x_{n}\right)\right|-w^{2}\left|x_{n}-NH\left(x_{n}\right)\right|,$$

where |•| is an element-wise absolute value operator. Intuitively, the larger *L*(**x**_*n*_|**w**) is, the more likely that **x**_*n*_ is correctly classified. Here we approach the distance ${\;\text{w}}^{2}|{\text{x}}_{n}-NM\left({\text{x}}_{n}\right)|$ and ${\text{w}}^{2}|{\text{x}}_{n}-NH\left({\text{x}}_{n}\right)|$ between the current sample to the most similar pattern in a probabilistic way, respectively, i.e., to estimate the probabilistic distribution by kernel method (Bishop, 2006; Sun et al., 2010) based on all the samples within corresponding set. Then the expectation of *L*(**x**_*n*_|**w**) will be equivalent as:

(2) $$\begin{array}{l} E\left[L\left(x_{n}|w\right)\right]=E\left[w^{2}\left|x_{n}-NM\left(x_{n}\right)\right|-w^{2}\left|x_{n}-NH\left(x_{n}\right)\right|\right]\\ \;=w^{2}(\sum_{x_{i}\in M}P\left(x_{i}=NM\left(x_{n}\right)|w\right)\left|x_{n}-x_{i}\right|\\ \;-\sum_{x_{i}\in H}P\left(x_{i}=NH\left(x_{n}\right)|w\right)\left|x_{n}-x_{i}\right|≜w^{2}{\bar{g}}_{n}, \end{array}$$

where *M* is the set of EEG spatial-temporal patterns with different class as **x**_*n*_, *H* includes those with the same class, *P*(**x**_*i*_ = *NM*(**x**_*n*_)|**w**) and *P*(**x**_*i*_ = *NH*(**x**_*n*_)|**w**) are the estimated probabilistic distributions of *NM*(**x**_*n*_) and *NH*(**x**_*n*_) by kernel method (Bishop, 2006; Sun et al., 2010).

In order to make the weight sparser, we add regularization penalty on vector **w**, and **w** is obtained by solving the optimization problem below:

(3) $$\underset{J\left(w\right)=\sum_{n=1}^{N}\log\left(1+exp\left(-w^{2}{\bar{g}}_{n}\right)\right)+\lambda {\left\|w\right\|}_{2}^{2}.}{{\text{min}}_{w}\text{J}(w),}$$

An iterative method is applied for obtaining the optimal value of **w**. First, we calculate the partial derivative for each element of **w**:

(4) $$\frac{\partial \text{J}\left(w\right)}{\partial w_{i}}=2\lambda w_{i}-2w_{i}\sum_{n=1}^{N}\frac{\exp\left(-\sum_{j}w_{j}^{2}{\bar{\text{g}}}_{n}\left(j\right)\right)}{1+exp\left(-\sum_{j}w_{j}^{2}{\bar{\text{g}}}_{n}\left(j\right)\right)}{\bar{g}}_{n}\left(i\right)\text{​}.$$

Then each element of **w** is initialized to 1. In each iteration, previously estimated **w** is first used to compute ${\bar{\text{g}}}_{n}$, then **w** is updated by the following rule:

(5) $$\begin{array}{c} \;w=w-\eta \nabla J\left(w\right)\\ =w-\eta \left(\lambda 1-\sum_{n=1}^{N}\frac{\exp\left(-\sum_{j}w_{j}^{2}{\bar{\text{g}}}_{n}(j)\right)}{1+\exp\left(-\sum_{j}w_{j}^{2}{\bar{\text{g}}}_{n}(j)\right)}{\bar{g}}_{n}\right)⊗w, \end{array}$$

where ⊗ is the Hadamard operator and η is the learning rate. When the iteration terminates at the optimal solution, we finally obtain the importance of EEG spatial-temporal features represented by the learned weight vector **w**^2^. In our work, the parameters in the LLB method were determined by 3-fold cross-validation using training set.

#### Classification algorithm and performance evaluation

In this study, we adopted the kernel support vector machine (SVM), which has been proved very effective in BCI classification problem (Kaper et al., 2004; Li et al., 2008; Rakotomamonjy and Guigue, 2008), to detect *single-trial* ERP for face identification. Given EEG spatial-temporal feature pattern **x**_*n*_ and the corresponding class label *y*_*n*_ (1 or −1 corresponding to target and no target face, respectively), the hyperplane could be obtained by optimizing the following problem:

(6) $$\begin{array}{l} \underset{\alpha }{\text{max}}\sum_{n=1}^{N}\alpha_{n}-\frac{1}{2}\sum_{n,m=1}^{N}\alpha_{n}\alpha_{m}y_{n}y_{m}K\left(x_{n},x_{m}\right),\\ \;s.t.\;0\leq \alpha_{n}\leq C,n=1,\ldots ,N,\\ \;\sum_{n=1}^{N}\alpha_{n}y_{n}=0, \end{array}$$

where *N* is the total number of the EEG spatial-temporal feature patterns, α_*n*_ is the Lagrangian multiplier. *K*(**x**_*n*_, **x**_*m*_) is the kernel function which implicitly maps the single-trial EEG spatial-temporal patterns to a high-dimensional feature space to make them more separable. The parameter *C* controls the balance between the classificaition accuracy in training set and good generalization. For the detail of the derivations of SVM, one can refer to (Cristianini Shawe-Taylor and Shawe-Taylor, 2000). Then the classification function is as follows:

(7) $$f\left(x\right)=\text{sgn}\left(\sum_{n=1}^{N}y_{n}\alpha_{n}\text{K}\left(x,x_{n}\right)+b\right),$$

where $b=y_{n}-\overset{N}{\underset{m=1}{\sum }}{y_{m}\alpha }_{m}\text{K}\left({\text{x}}_{n},{\text{x}}_{m}\right)$ for any **x**_*n*_ with 0 < α_*n*_ < *C*.

In our work, kernel SVM (RBF-SVM) implemented by LIBSVM (Chang and Lin, 2011) was used to perform the target/nontarget EEG classification nonlinearly. The kernel width σ and the regularization parameter *C* were determined by a 3-fold cross validation.

In the current study, we used the area under the receiver operating characteristic (ROC) curve (AUC) as the performance evaluation criterion (Fawcett, 2004). The ROC curve depicts the tradeoff between the FPR and the TPR. The AUC's value is between 0 and 1, and a larger AUC means a better classification performance (Fawcett, 2004).

## Results

In this section, we first examine the ERP waves elicited by the target and non-target faces. Then we adopt the LLB method to extract the important spatial-temporal features, and validate the feature importance by the target identification accuracy using feature subset. The spatial-temporal importance distribution by proposed method is compared with other three feature extraction methods, including FC, SVMRFE, and mutual information (MI). The sparse spatial-temporal distribution of important ERP features is identified, particularly with the early temporal course and the spatial electrode distribution for the task of rapid face identification.

### Grand-average ERPs

**Figure 3:**
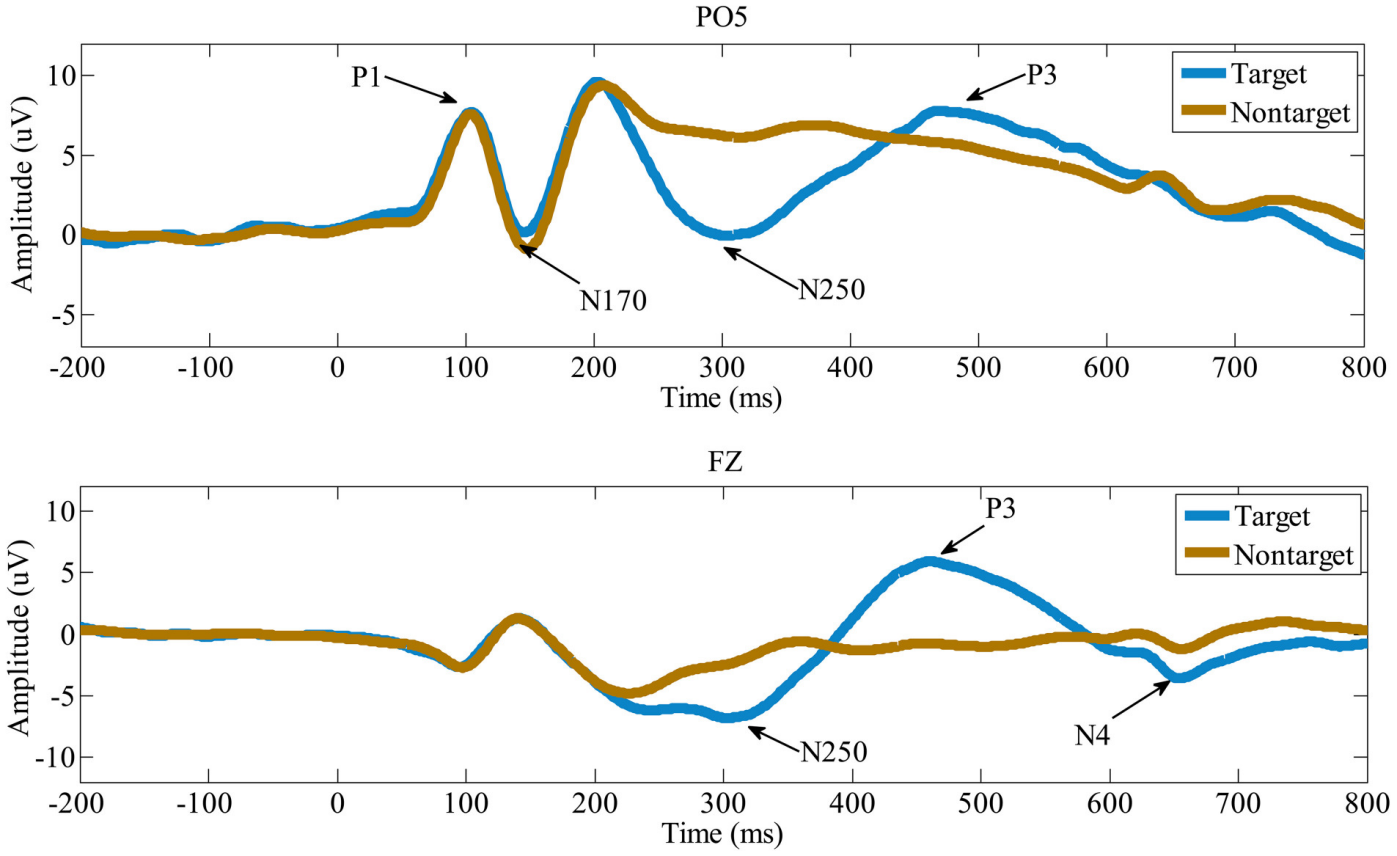
The grand-average ERPs in electrode PO5 and FZ. Compared to the non-target faces, the target faces evoke distinct N250, P3, and N4.

Figure 3 shows the ERPs averaged across the all 15 participants in electrode PO5 and FZ. X-axis is the time (ms) and y-axis is the ERP amplitude (uV). From the ERP waves in Figure 3, we can see that P1 component is elicited at about 100 ms post face image presented, followed by a face-specific N170 at about 150 ms. These early components (including the P1 and the N170) elicited by target and non-target faces are highly similar in morphology. While compared to nontarget faces, the target faces elicit a N250 component lasting from about 240 to 390 ms, followed by P3 (390~590 ms) and N4 (600~700 ms). These results indicate that the early components may be not linked to face identification directly, the subsequent components starting with the N250, are associated with face identification and would play important roles in the single-trial EEG classification.

### Spatial-temporal feature extraction

**Figure 4:**
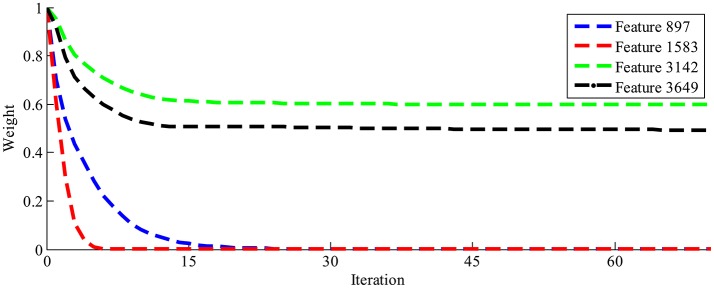
The time evolution of four feature weights learned by the LLB method.

The LLB feature extraction method is adopted to evaluate the importance of EEG spatial-temporal features for face identification. The LLB method endows a weight to each spatial-temporal feature to represent its importance. As an iterative approach, the LLB method updates the weights for all features in each iteration. Figure 4 shows an example of the time evolution of the feature weights learned by the LLB method. We can see that as iterations go, the weights of feature 3,142 and feature 3,649 are still large while those of feature 897 and feature 1,583 drop down to 0 quickly within 30 iterations. For all participants, we observe that the weights of spatial-temporal features usually converge within 50 iterations.

**Figure 5:**
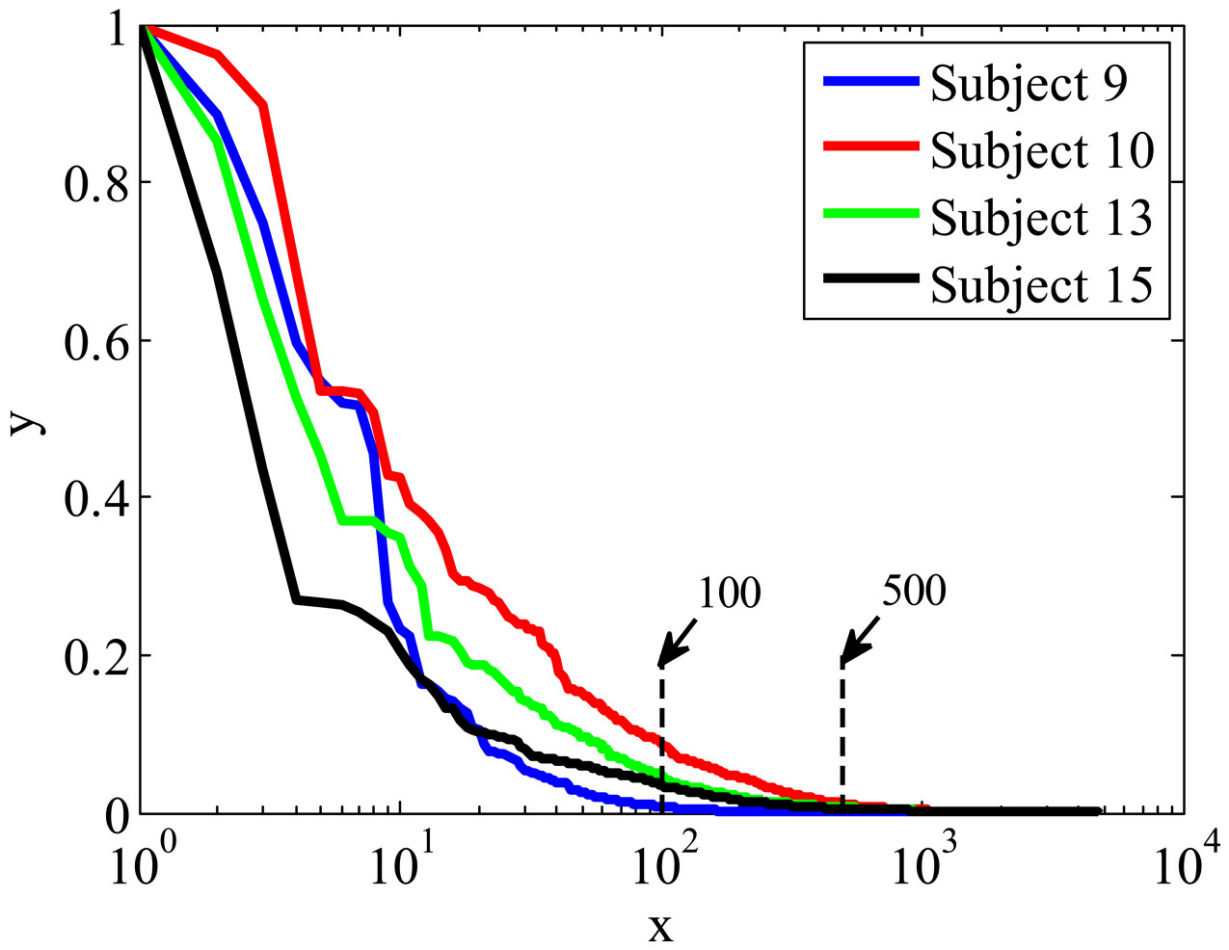
The importance weights in descending order calculated by LLB method for four participants.

Figure 5 shows the importance weights calculated by LLB method for four participants, the spatial-temporal features are ranked according to their importance in descending order. X is the feature index after ranking in logarithm scale, y is the feature importance. We can see that, among all 4,800 features, the ones ranked within top 100 have relative big weights, while the weights of the features ranked after 500 are close to 0. These results indicate that EEG signals contain many features with low weights, using LLB method to extract features with big weights can greatly reduce the dimension of ERP features.

**Figure 6:**
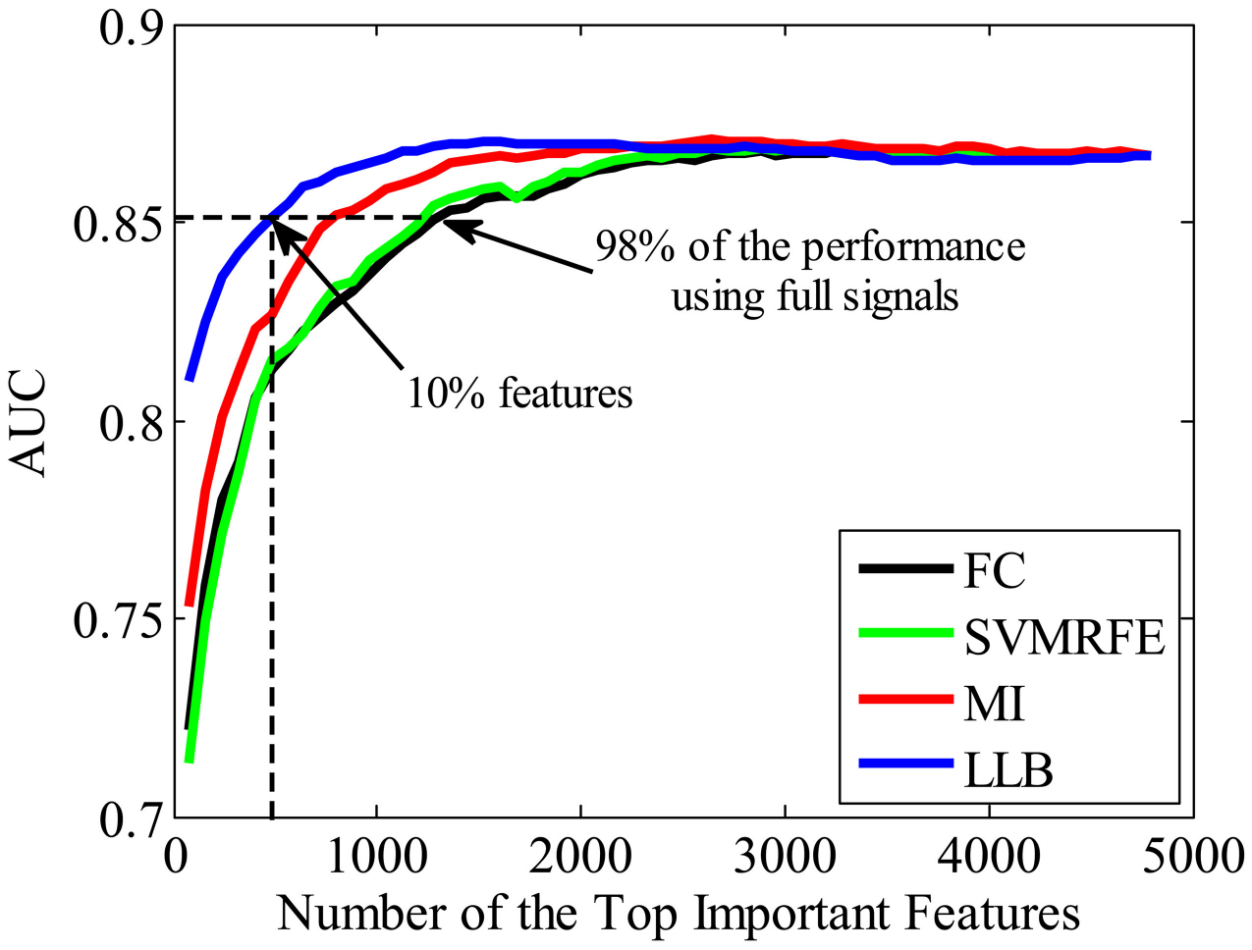
The average single-trial ERP detection performance over all participants as a function of the number of important spatial-temporal features used. Four feature extraction methods are used to evaluate the feature importance, including the fisher criterion (FC) (black), support vector machine recursive feature elimination (SVMRFE) (green), mutual information (MI) (red), and local-learning-based (LLB) method (blue).

To validate the important ERP features extracted by the proposed LLB method, we evaluate the face identification performance by single-trial ERP detection using only the important feature subset. For comparison, other three feature extraction algorithms are also adopted, including the FC (Krusienski et al., 2008; D'croz-Baron et al., 2012; Guo et al., 2015), SVMRFE (Guyon et al., 2002; Hidalgo-Muñoz et al., 2013), and mutual information (MI) (Shahriari and Erfanian, 2011; Ang et al., 2012). The importance of spatial-temporal features is evaluated by the above 4 methods, and features are ranked according to their importance in descending order. Figure 6 shows the statistical single-trial ERP detection performance averaged across participants as a function of the number of important features for the four methods. X-axis is the feature number and the y-axis is the classification performance (AUC) using the same kernel SVM method but with different feature subsets by four methods. We can see that the performance increases as important features involved in the classification. And with the same number of the important features, those extracted by the LLB method always obtain the best classification performance before the performance converges. We also find that the average AUC achieves more than 0.851 (98% of the performance using the full signals, 0.867) with only the most important 480 features (10% of the total features). These results indicate LLB method can effectively identify the important spatial-temporal features, and the most important 480 features hold the majority of discriminant information for single-trial face identification.

**Figure 7:**
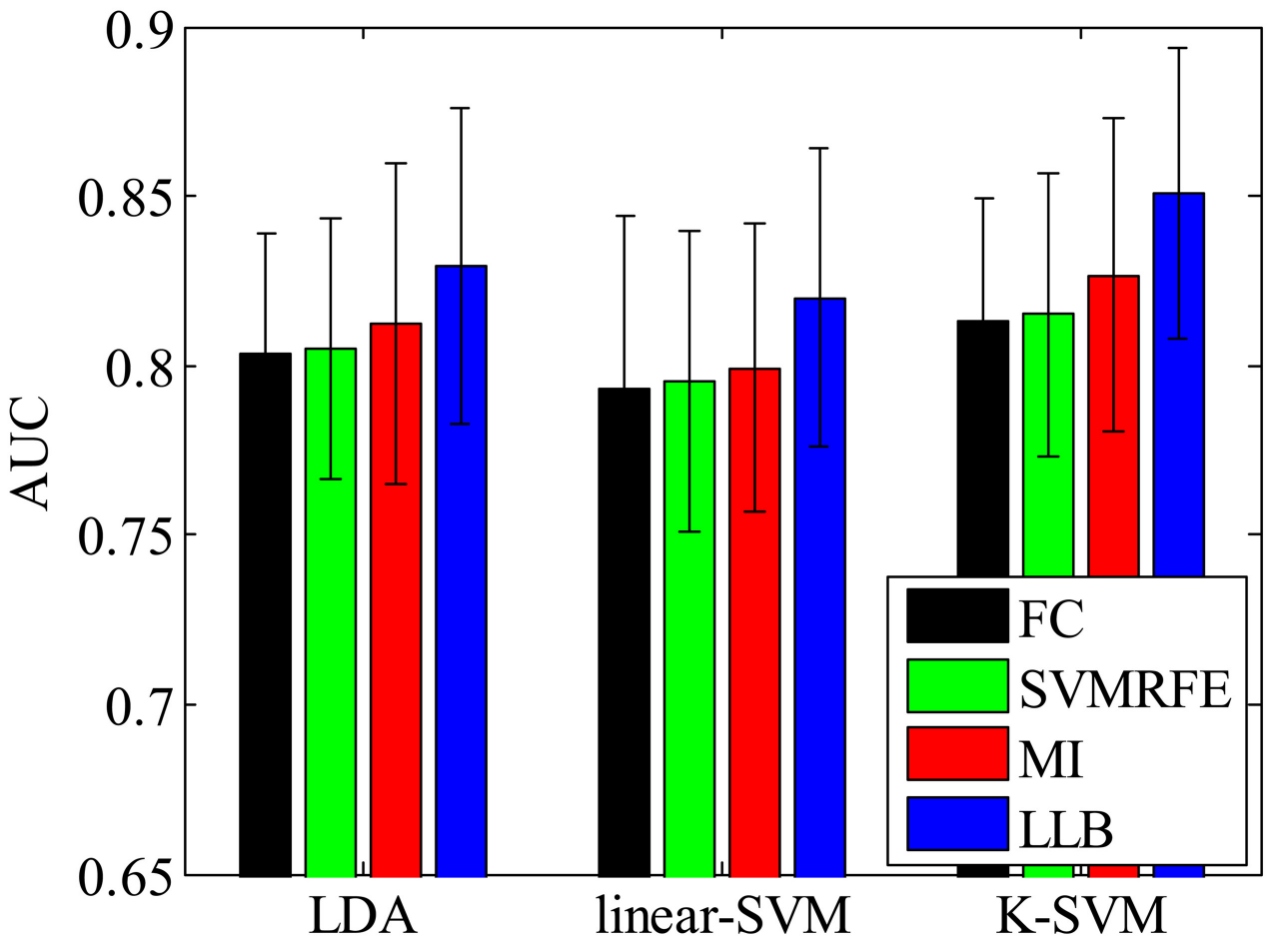
The single-trial ERP detection performance using the top 480 important features by fisher criterion (FC) (black), support vector machine recursive feature elimination (SVMRFE) (green), mutual information (MI) (red), and local-learning-based (LLB) method (blue).

We also want to make sure that the extracted spatial-temporal feature subset is consistently important even using different classifiers on single-trial ERP detection. Figure 7 shows the performance (AUC) using LDA (Bishop, 2006), linear SVM (linear-SVM), and kernel SVM (K-SVM), with the top 10% important features extracted by FC, SVMRFE, MI, and LLB method, respectively. We can see that, the important features selected by the LLB method always achieve the best performance across different classifiers. This is because LLB method optimizes the importance in the feature space, where the nonlinear EEG data structure is decomposed and the local information of single trial ERP features is preserved. Therefore, the selected important features by LLB method doesn't rely on the specific classification model, the important features can constantly achieve the best performance using different classifiers.

### Spatial-temporal feature analysis

**Figure 8:**
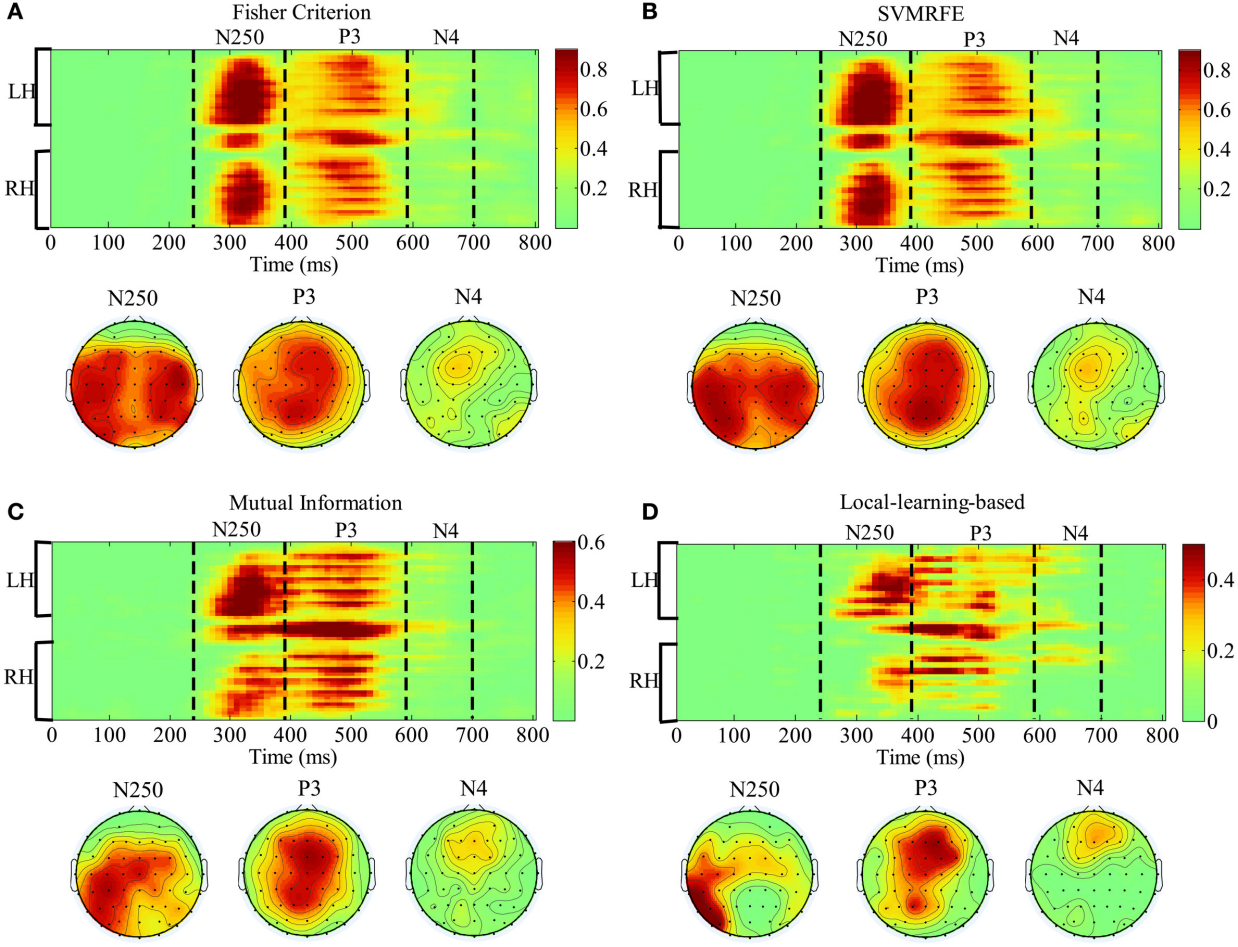
The representation of the spatial-temporal distribution of important features by fisher criterion (FC) **(A)** support vector machine recursive feature elimination (SVMRFE) **(B)** mutual information (MI) **(C)** and local-learning-based (LLB) method **(D)**. Time (ms) is on the x-axis and electrodes are on the y-axis. Electrodes are organized in the following order: left hemisphere (LH), midline electrodes, right hemisphere (RH). The time intervals of different ERP components are separated by dash lines.

In this section, we analyze the spatial-temporal distribution of important features by FC, SVMRFE, MI and LLB method. We investigate the feature subsets which achieve 98% of the performance using full signals, shown as the dash horizontal line also in Figure 6. Here FC and SVMRFE need 1,280 (26.7%) features, MI needs 800 (16.7%) features, and LLB method only needs 480 (10%) important features. We first define a vector function *B*_*j*_(**F**), *j* is the participant index, **F** represents the ERP feature vector (the dimension of **F** is 4800). When the feature *F*_*i*_ (*i* = 1, …, 4, 800) is selected as important feature for participant *j*, the value of i^th^ item in *B*_*j*_(**F**) equals to 1, zero otherwise. The mean of *B*_*j*_(**F**) across 15 participants is visualized as the probabilistic distribution of the important features in top plot of Figure 8. Time (ms) is on the x-axis and electrodes are on the y-axis. Electrodes are organized in the following order: left hemisphere (LH), midline electrodes, right hemisphere (RH). The time intervals of different ERP components are separated by vertical dash lines. Figure 8 also shows the scalp topographies of important features in N250, P3, and N4 in bottom plot. We can see that the vast majority of important features are distributed in the time windows of N250, P3, and N4, but not in the earlier components, these results suggest the N250, P3, and N4 are related to face identification directly, the N250 is the earliest component linked to face identification. Among all the feature extraction methods, LLB method demonstrates the sparsest distribution. And important features by LLB methods are more laterally distributed toward the left hemisphere than other methods, especially during the N250 time window where important features are widely distributed over the left central and left parietal-occipital regions. During the P3 time window, the important features are mainly distributed in the midline electrodes, which is consistent with previous studies on P3 distribution (Polich, 2007). In addition, important features are distributed in the frontal regions in the late N4 time window.

**Figure 9:**
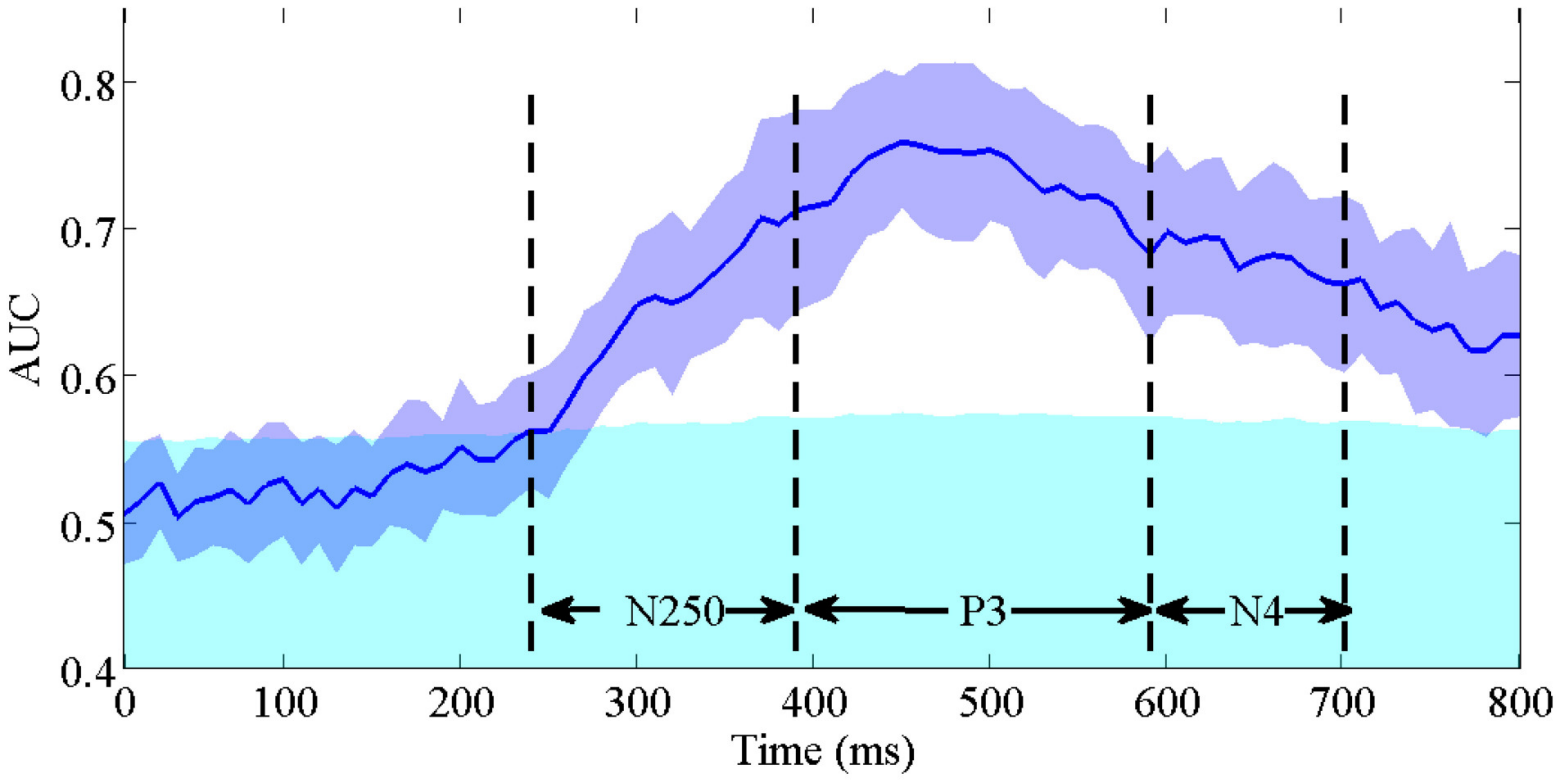
The averaged single-trial ERP detection performance (AUC) across participants as a function of temporal component every 10 ms. The purple blue area represents the standard derivation of classification performance. The cyan area represents the upper bound of the 95% confidence interval for chance level. The time intervals of N250, P3, and N4 are indicated by dash lines.

To validate the contribution of the early N250 component indicated by the above spatial-temporal important feature distribution, we conduct single-trial ERP detection using the temporal components of the signals within every 10 ms. Figure 9 shows the averaged performance (AUC) across participants as a function of temporal component of the EEG used. X-axis is the time (ms) and the y-axis is the classification performance (AUC). The purple blue area represents the standard derivation of classification performance, the cyan area represents the upper bound of the 95% confidence interval (calculated by a permutation procedure; Rieger et al., 2008) for chance level. The time windows of N250, P3, and N4 are indicated by dash lines. We can see that the classification performance firstly exceeds the upper 95% confidence interval (the cyan area) as the ERP features in the N250 time window are used in classification. The highest performance is obtained within the P3 time window. Afterwards the performance drops using only N4 component. These results demonstrate that N250 is the earliest temporal component contributing to the single-trial ERP detection in the task of rapid face identification.

**Figure 10:**
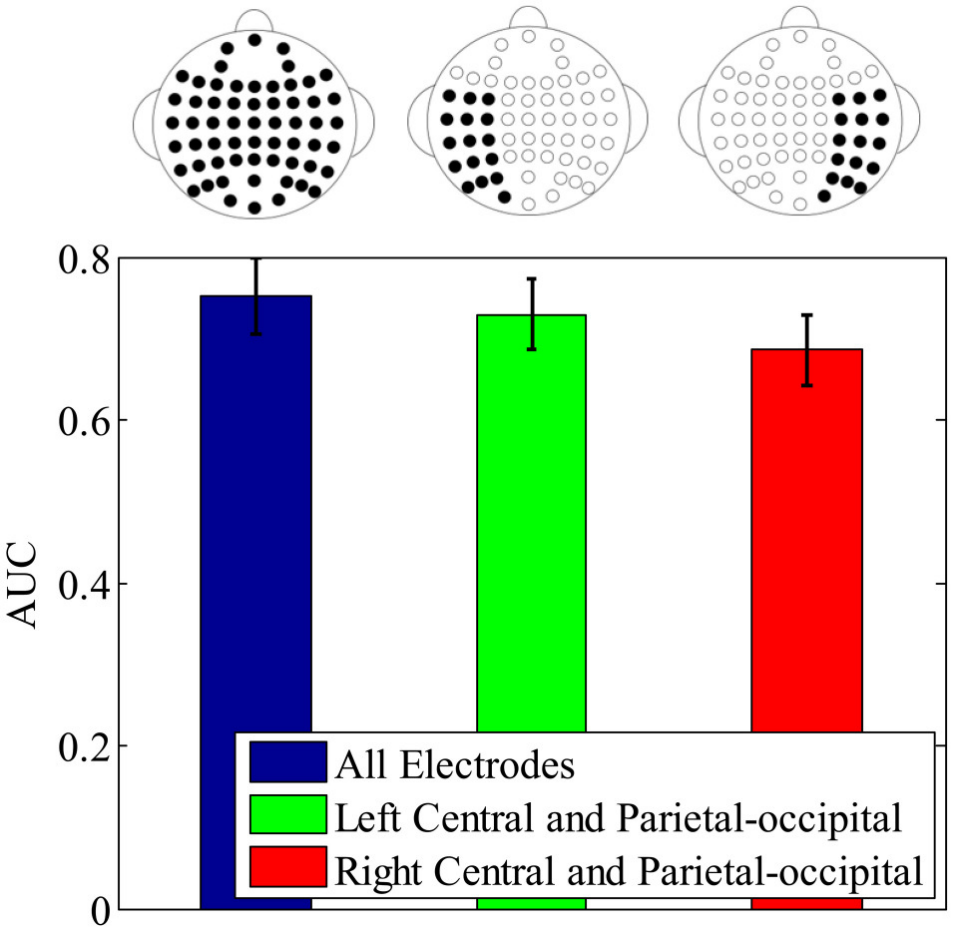
The single-trial ERP detection performance using N250 component from all electrodes (blue error bar), the left central and left parietal-occipital electrodes (green error bar), right central and right parietal-occipital electrodes (red error bar), respectively. The electrodes used for classification are depicted by the electrode topographies on the top plot.

To further validate the lateral spatial distribution of earliest component N250, which is indicated by the above important feature distribution, the single-trial ERP detection performance using N250 component in *original* signals is compared with all electrodes, the left central and left parietal-occipital electrodes, and the corresponding right central and right parietal-occipital electrodes, respectively. Figure 10 shows the averaged single-trial ERP detection performance (AUC) across participants using different electrode subsets. The performance using the N250 component from the left central and left parietal-occipital electrodes is significantly higher than the performance using the corresponding right central and right parietal-occipital electrodes (0.7296 ± 0.0434 vs. 0.6855 ± 0.0431; one-tailed paired *t*-test, *p* = 8.1270e-004), and achieves the 97.01% performance of the full electrodes. These results demonstrate that, the N250 has a left-hemisphere advantage on single-trial ERP detection in the task of face identification. Using the temporal component as early as the N250 (~310 ms), the time required to identify individual target faces is greatly reduced comparing to the one using the temporal information until P3 (~480 ms), not mentioning the button press movement (~653 ms). And the lateral importance distribution of the N250 in the left hemisphere indicates the potential of using fewer electrodes for single-trial detection in the task of rapid face identification.

## Conclusion and discussion

Event related potential (ERP) reflects the process of brain cognitive activity, and can be introduced to BCI real-time applications to represent the subject's intention. Due to the low signal-to-noise ratio (SNR) of EEG, most ERP studies are based on grand-averaging (Luck, 2014). In the real-time tasks as rapid face identification, all the targets needed to be retrieved may appear only once, and there is no knowledge of target label for trial-averaging. Therefore, single-trial ERP detection is necessary. In addition, single-trial analysis increases the efficiency in identifying target faces, and reduces the fatigue of subjects. Extracting the important temporal-spatial features that contribute to single-trial ERP detection helps to design an efficient BCI platform of the rapid face identification, in term of the fast identification speed using the early temporal component and less computation using only partial electrodes.

In this paper, we propose to implement a LLB method to investigate the importance of spatial-temporal features for single-trial ERP detection in rapid face identification. As a nonlinear method, the LLB method decomposes the nonlinear EEG signal into a feature space to preserve the structure of the signal locally (Sun et al., 2010). The importance of the EEG spatial-temporal features is obtained through the optimization of the clustering within the same class (all targets or all non-targets) but further away from another class. Without priori hypothesis, the computation is based on the EEG data distribution and does not depend on the ERP detection methods. In addition, the proposed LLB method introduces a weight decay regularization for sparse weights, which makes the weights of irrelevant features close to zero. Here we compare LLB method with three existing feature extraction methods, including FC, SVMRFE, and mutual information (MI). FC and SVMRFE have linear assumption, which cannot capture the nonlinear structure of the EEG signal, and the extracted features depend on the ERP detection models. MI is data-driven and free of modeling, but the extracted features may not be sparse. Results show that the LLB method identifies important spatial-temporal features with only 480 selected important features (10% of the total features) achieving more than 98% performance of the full features. Comparing to other 3 methods, the important features are distributed more sparsely, and sufficiently good in terms of single-trial ERP detection across different classifiers.

We analyze the distribution of important spatial-temporal features, and find vast majority of important features are distributed in the time windows of N250, P3, and N4. The results show that the single-trial ERP detection performance starts to significantly exceed the guessing interval in the N250 time window. It suggests the N250, which emerges around 240 ms after stimulus onset, is the earliest ERP component that contributes to the single-trial ERP detection in the task of face identification. Previous ERP studies have suggested that the P1 is linked to the processing of low-level visual features (Zheng et al., 2012), and the N170 reflects the face-specific structural encoding processes (Gosling and Eimer, 2011). In our task, P1 and N170 occur in both target and non-target cases due to the fact all the images are faces, consequently they do not contribute directly to the task of face identification. Our finding of the early component N250 is consistent with the results in (Gosling and Eimer, 2011; Zheng et al., 2012), where N250 is believed to reflect the early stages where a perceptual face representation is compared to previously known faces, but our work advances more in the validation using single-trial ERP detection instead of conventional grand-averaging. From an applications perspective, we demonstrate that single-trial ERP could be detected almost immediately after N250 occurs, which benefits to building a much faster BCI-based face identification system than button press.

Another interesting finding concerns the laterality of the N250. We find the important N250 components are more laterally distributed and biased toward the left hemisphere during the time window from 240 to 390 ms (see Figure 8). In comparison, the P3 does not show significant lateral distribution (Supplementary Figure 1). And the subsequent single-trial ERP detection using only left hemisphere electrodes of N250 component confirms this lateral distribution advantage. Some previous ERP studies using trial-averaging have also reported similar left-hemisphere bias of the N250. In (Nessler et al., 2005), Nessler et al. reported that the N250 differentiated familiar faces from unfamiliar faces at left parietal-occipital, left frontal, and central electrodes, while it was not found over right hemisphere. In the face identification experiment conducted by Huang et al., the N250 elicited by familiar targets was lateralized to the left occipitotemporal electrodes (Huang et al., 2017). Some brain imaging studies also find similar left-lateralized face identification process. A PET study conducted by Gornotempini et al. showed that compared to unfamiliar faces, familiar faces activated the areas spreading from the left anterior temporal to the left temporo-parietal regions (Gornotempini et al., 1998). Sugiura et al. reported activation in left medial temporal regions during recognition of familiar faces (Sugiura et al., 2001). Activations of the left hippocampus and left fusiform gyrus were observed for familiar as compared to unfamiliar faces in an fMRI study conducted by Eger et al. (2005). Previous studies have suggested that right hemisphere stores and processes visual stimuli in image-dependent manner, whereas the left hemisphere stores and processes visual stimuli in a more abstractive, image-independent manner (Marsolek, 1999; Cooper et al., 2007; Meng et al., 2012). Therefore, we speculate the laterality of N250 in our study reflects the advantage of left hemisphere in identifying the target faces across different viewpoints and lighting conditions, even in the presence of partial occlusion during our experiment. In addition, considering the hypothesis that the N250 is generated in the fusiform gyri (Schweinberger et al., 2002; Tanaka et al., 2006), we speculate that the left fusiform gyrus might be associated with the signal during the N250 time window (240–390 ms after stimulus onset) in our rapid face identification task. To locate the brain sources of the N250 more precisely, some source localizations or brain imaging techniques (e.g., PET and fMRI) could be applied in the future work.

In summary, we propose to adopt a LLB feature extraction method to investigate the importance of spatial-temporal components for single-trial ERP detection in a task of rapid face identification. Comparing with other methods, the LLB method uses fewer and sparser features to achieve the comparable performance as the whole features across different detection methods. The interesting finding is that the N250 is the earliest temporal component with laterally distribution in term of contribution to single-trial ERP detection in rapid face identification. This is validated by the fact that using only the left N250 component over-performs the right N250 in the face identification task. Our finding is beneficial in building a fast (using the early N250) and efficient (using fewer electrodes) BCI system for rapid face identification.

## Ethics statement

The experiments were conducted in accordance with the Declaration of Helsinki. The experiments in our study were approved by the ethics committee of Zhejiang University.

## Author contributions

LJ, YiW and YueW contributed to design of the idea. LJ, YunW, and BC contributed in the data collection. LJ, YunW, BC, YiW, and YueW contributed in data processing and results analysis. LJ and YiW contributed in developing the manuscript.

### Conflict of interest statement

The authors declare that the research was conducted in the absence of any commercial or financial relationships that could be construed as a potential conflict of interest.

## References

[B1] Ang K. K. Chin Z. Y. Zhang H. Guan C. (2012). Mutual information-based selection of optimal spatial–temporal patterns for single-trial EEG-based BCIs. Pattern Recog. 45, 2137–2144. 10.1016/j.patcog.2011.04.018

[B2] Belitski A. Farquhar J. Desain P. (2011). P300 audio-visual speller. J. Neural Eng. 8:025022. 10.1088/1741-2560/8/2/025022 21436523

[B3] Bentin S. Deouell L. Y. (2000). Structural encoding and identification in face processing: ERP evidence for separate mechanisms. Cogn. Neuropsychol. 17, 35–55. 10.1080/026432900380472 20945170

[B4] Bishop C. M. (2006). Pattern Recognition. Berlin: Springer.

[B5] Cai B. Xiao S. Jiang L. Wang Y. Zheng X. (2013). A rapid face recognition BCI system using single-trial ERP, in Proceedings of the 6th International IEEE/EMBS Conference on Neural Engineering (NER) (San Diego, CA: IEEE), 89–92.

[B6] Chang C.-C. Lin C.-J. (2011). LIBSVM: a library for support vector machines. ACM Trans. Intell. Syst. Technol. 2, 1–27. 10.1145/1961189.1961199

[B7] Chen B. C. Chen Y. Y. Kuo Y. H. Hsu W. H. (2013). Scalable face image retrieval using attribute-enhanced sparse codewords. IEEE Trans. Multimed. 15, 1163–1173. 10.1109/TMM.2013.2242460

[B8] Chen W.-D. Zhang J.-H. Zhang J.-C. Li Y. Qi Y. Su Y. (2010). A P300 based online brain-computer interface system for virtual hand control. J. Zhejiang Univ. Sci. 11, 587–597. 10.1631/jzus.C0910530

[B9] Cooper T. J. Harvey M. Lavidor M. Schweinberger S. R. (2007). Hemispheric asymmetries in image-specific and abstractive priming of famous faces: evidence from reaction times and event-related brain potentials. Neuropsychologia 45, 2910–2921. 10.1016/j.neuropsychologia.2007.06.005 17663008

[B10] Cristianini N. Shawe-Taylor J. (2000). An Introduction to Support Vector Machines. Cambridge, UK: Cambridge University press.

[B11] Curran T. Hancock J. (2007). The FN400 indexes familiarity-based recognition of faces. Neuroimage 36, 464–471. 10.1016/j.neuroimage.2006.12.016 17258471 PMC1948028

[B12] D'croz-Baron D. Ramirez J. M. Baker M. Alarcon-Aquino V. Carrera O. (2012). A BCI motor imagery experiment based on parametric feature extraction and fisher criterion, in Proceedings of the 22nd International Conference on Electrical Communications and Computers (CONIELECOMP) (Cholula: IEEE), 257–261.

[B13] Donchin E. Spencer K. M. Wijesinghe R. (2000). The mental prosthesis: assessing the speed of a P300-based brain-computer interface. IEEE Trans. Rehabil. Eng. 8, 174–179. 10.1109/86.847808 10896179

[B14] Eger E. Schweinberger S. Dolan R. Henson R. (2005). Familiarity enhances invariance of face representations in human ventral visual cortex: fMRI evidence. Neuroimage 26, 1128–1139. 10.1016/j.neuroimage.2005.03.010 15961049

[B15] Eimer M. (2000). Event-related brain potentials distinguish processing stages involved in face perception and recognition. Clin. Neurophysiol. 111, 694–705. 10.1016/S1388-2457(99)00285-0 10727921

[B16] Fawcett T. (2004). ROC graphs: notes and practical considerations for researchers. Mach. Learn. 31, 1–38.

[B17] Gao Y. Qi Y. (2005). Robust visual similarity retrieval in single model face databases. Pattern Recog. 38, 1009–1020. 10.1016/j.patcog.2004.12.006

[B18] Gazzaniga M. S. Ivry R. B. Mangun G. R. Steven M. S. (2009). Cognitive Neuroscience: the Biology of the Mind. New York, NY: W. W. Norton.

[B19] George N. Dolan R. J. Fink G. R. Baylis G. C. Russell C. Driver J. (1999). Contrast polarity and face recognition in the human fusiform gyrus. Nat. Neurosci. 2, 574–580. 10.1038/9230 10448224

[B20] Gornotempini M. L. Price C. J. Josephs O. Vandenberghe R. Cappa S. F. Kapur N. (1998). The neural systems sustaining face and proper-name processing. Brain 121, 2103–2118. 10.1093/brain/121.11.2103 9827770

[B21] Gosling A. Eimer M. (2011). An event-related brain potential study of explicit face recognition. Neuropsychologia 49, 2736–2745. 10.1016/j.neuropsychologia.2011.05.025 21679721

[B22] Guo S. Lin S. Huang Z. (2015). Feature extraction of P300s in EEG signal with discrete wavelet transform and fisher criterion, in Proceedings of the 8th International Conference on Biomedical Engineering and Informatics (BMEI) (Shenyang, China: IEEE), 200–204.

[B23] Guyon I. Weston J. Barnhill S. Vapnik V. (2002). Gene selection for cancer classification using support vector machines. Mach. Learn. 46, 389–422. 10.1023/A:1012487302797

[B24] Han J. Chen C. Shao L. Hu X. Han J. Liu T. (2015a). Learning computational models of video memorability from fMRI brain imaging. IEEE Trans. Cybern. 45, 1692–1703. 10.1109/TCYB.2014.2358647 25314715

[B25] Han J. Ji X. Hu X. Guo L. Liu T. (2015b). Arousal recognition using audio-visual features and FMRI-based brain response. IEEE Trans. Affect. Comput. 6, 337–347. 10.1109/TAFFC.2015.2411280

[B26] Han J. Ji X. Hu X. Zhu D. Li K. Jiang X.. (2013). Representing and retrieving video shots in human-centric brain imaging space. IEEE Trans. Image Process. 22, 2723–2736. 10.1109/TIP.2013.2256919 23568507 PMC3984391

[B27] Haxby J. V. Hoffman E. A. Gobbini M. I. (2002). Human neural systems for face recognition and social communication. Biol. Psychiatry 51, 59–67. 10.1016/S0006-3223(01)01330-0 11801231

[B28] Hidalgo-Muñoz A. R. López M. M. Galvao-Carmona A. Pereira A. T. Santos I. M. Vázquez-Marrufo M.. (2014). EEG study on affective valence elicited by novel and familiar pictures using ERD/ERS and SVM-RFE. Med. Biol. Eng. Comput. 52, 149–158. 10.1007/s11517-013-1126-6 24257836

[B29] Hidalgo-Muñoz A. R. López M. M. Santos I. M. Pereira A. T. Vázquez-Marrufo M. Galvao-Carmona A. (2013). Application of SVM-RFE on EEG signals for detecting the most relevant scalp regions linked to affective valence processing. Expert Syst. Appl. 40, 2102–2108. 10.1016/j.eswa.2012.10.013

[B30] Hoffmann U. Vesin J. M. Ebrahimi T. Diserens K. (2008). An efficient P300-based brain–computer interface for disabled subjects. J. Neurosci. Methods 167, 115–125. 10.1016/j.jneumeth.2007.03.005 17445904

[B31] Huang W. Wu X. Hu L. Wang L. Ding Y. Qu Z. (2017). Revisiting the earliest electrophysiological correlate of familiar face recognition. Int. J. Psychophysiol. 120, 42–53. 10.1016/j.ijpsycho.2017.07.001 28684327

[B32] Jon T. Laurie G. Horne J. H. Paul W. (2011). Real-time measurement of face recognition in rapid serial visual presentation. Front. Psychol. 2:42 10.3389/fpsyg.2011.00042 21716601 PMC3110906

[B33] Kai B. Schulze S. Stodieck S. R. G. (1995). Scalp topography and analysis of intracranial sources of face-evoked potentials. Exp. Brain Res. 104, 135–143. 7621932 10.1007/BF00229863

[B34] Kanwisher N. Mcdermott J. Chun M. M. (1997). The fusiform face area: a module in human extrastriate cortex specialized for face perception. J. Neurosci. 17, 4302–4311. 9151747 10.1523/JNEUROSCI.17-11-04302.1997 PMC6573547

[B35] Kaper M. Meinicke P. Grossekathoefer U. Lingner T. Ritter H. (2004). BCI competition 2003-data set IIb: support vector machines for the P300 speller paradigm. IEEE Trans. Biomed. Eng. 51, 1073–1076. 10.1109/TBME.2004.826698 15188881

[B36] Keenan J. P. Nelson A. O'connor M. Pascualleone A. (2001). Self-recognition and the right hemisphere. Nature 409, 305. 10.1038/35053167 11201730

[B37] Krusienski D. J. Sellers E. W. Mcfarland D. J. Vaughan T. M. Wolpaw J. R. (2008). Toward enhanced P300 speller performance. J. Neurosci. Methods 167, 15–21. 10.1016/j.jneumeth.2007.07.017 17822777 PMC2349091

[B38] Kuskowski M. A. Pardo J. V. (1999). The role of the fusiform gyrus in successful encoding of face stimuli. Neuroimage 9, 599–610. 10.1006/nimg.1999.0442 10334903

[B39] Li Y. Guan C. Li H. Chin Z. (2008). A self-training semi-supervised SVM algorithm and its application in an EEG-based brain computer interface speller system. Pattern Recog. Lett. 29, 1285–1294. 10.1016/j.patrec.2008.01.030

[B40] Li Y. Long J. Yu T. Yu Z. Wang C. Zhang H.. (2010). An EEG-based BCI system for 2-D cursor control by combining Mu/Beta rhythm and P300 potential. IEEE Trans. Biomed. Eng. 57, 2495–2505. 10.1109/TBME.2010.2055564 20615806

[B41] Liu S. Meng J. Zhao X. Yang J. He F. Qi H. (2014). Cross-task emotion recognition using EEG measures: first step towards practical application. Instrumentation 1, 17–24. 10.15878/j.cnki.instrumentation.2014.03.002

[B42] Loffler G. Yourganov G. Wilkinson F. Wilson H. R. (2005). fMRI evidence for the neural representation of faces. Nat. Neurosci. 8, 1386–1390. 10.1038/nn1538 16136037

[B43] Luck S. J. (2014). An Introduction to the Event-Related Potential Technique. Cambridge, MA: MIT press.

[B44] Marsolek C. J. (1999). Dissociable neural subsystems underlie abstract and specific object recognition. Psychol. Sci. 10, 111–118. 10.1111/1467-9280.00117

[B45] Mccarthy G. Puce A. Gore J. C. Allison T. (1997). Face-specific processing in the human fusiform gyrus. J. Cogn. Neurosci. 9, 605–610. 10.1162/jocn.1997.9.5.605 23965119

[B46] Meng M. Cherian T. Singal G. Sinha P. (2012). Lateralization of face processing in the human brain. Proc. Royal Soc. B Biol. Sci. 279, 2052–2061. 10.1098/rspb.2011.1784 22217726 PMC3311882

[B47] Nessler D. Mecklinger A. Penney T. B. (2005). Perceptual fluency, semantic familiarity and recognition-related familiarity: an electrophysiological exploration. Cogn. Brain Res. 22, 265–288. 10.1016/j.cogbrainres.2004.03.023 15653299

[B48] Piccione F. Priftis K. Tonin P. Vidale D. Furlan R. Cavinato M. (2008). Task and stimulation paradigm effects in a P300 brain computer interface exploitable in a virtual environment: a pilot Study. Psychnol. J. 6, 99–108.

[B49] Picton T. Bentin S. Berg P. Donchin E. Hillyard S. Johnson R.. (2000). Guidelines for using human event-related potentials to study cognition: recording standards and publication criteria. Psychophysiology 37, 127–152. 10.1111/1469-8986.3720127 10731765

[B50] Polich J. (2007). Updating P300: an integrative theory of P3a and P3b. Clin. Neurophysiol. 118, 2128–2148. 10.1016/j.clinph.2007.04.019 17573239 PMC2715154

[B51] Rakotomamonjy A. Guigue V. (2008). BCI competition III: dataset II-ensemble of SVMs for BCI P300 speller. IEEE Trans. Biomed. Eng. 55, 1147–1154. 10.1109/TBME.2008.915728 18334407

[B52] Rieger J. W. Reichert C. Gegenfurtner K. R. Noesselt T. Braun C. Heinze H. J.. (2008). Predicting the recognition of natural scenes from single trial MEG recordings of brain activity. Neuroimage 42, 1056–1068. 10.1016/j.neuroimage.2008.06.014 18620063

[B53] Rossion B. Campanella S. Gomez C. M. Delinte A. Debatisse D. Liard L.. (1999). Task modulation of brain activity related to familiar and unfamiliar face processing: an ERP study. Clin. Neurophysiol. 110, 449–462. 10.1016/S1388-2457(98)00037-6 10363769

[B54] Rossion B. Dricot L. Devolder A. Bodart J. M. Crommelinck M. De Gelder B.. (2000). Hemispheric asymmetries for whole-based and part-based face processing in the human fusiform gyrus. J. Cogn. Neurosci. 12, 793–802. 10.1162/089892900562606 11054921

[B55] Schweinberger S. R. Pickering E. C. Jentzsch I. Burton A. M. Kaufmann J. M. (2002). Event-related brain potential evidence for a response of inferior temporal cortex to familiar face repetitions. Cogn. Brain Res. 14, 398–409. 10.1016/S0926-6410(02)00142-8 12421663

[B56] Sellers E. W. Krusienski D. J. Mcfarland D. J. Vaughan T. M. Wolpaw J. R. (2006). A P300 event-related potential brain–computer interface (BCI): the effects of matrix size and inter stimulus interval on performance. Biol. Psychol. 73, 242–252. 10.1016/j.biopsycho.2006.04.007 16860920

[B57] Shahriari Y. Erfanian A. (2011). A mutual information based channel selection scheme for P300-based brain computer interface, in Proceedings of the 5th International IEEE/EMBS Conference on Neural Engineering (NER) (Cancun, IEEE), 434–437.

[B58] Sugiura M. Kawashima R. Nakamura K. Sato N. Nakamura A. Kato T.. (2001). Activation reduction in anterior temporal cortices during repeated recognition of faces of personal acquaintances. Neuroimage 13, 877–890. 10.1006/nimg.2001.0747 11304083

[B59] Sun Y. Todorovic S. Goodison S. (2010). Local-learning-based feature selection for high-dimensional data analysis. IEEE Trans. Pattern Anal. Mach. Intell. 32, 1610–1626. 10.1109/TPAMI.2009.190 20634556 PMC3445441

[B60] Tanaka J. W. Curran T. Porterfield A. L. Collins D. (2006). Activation of preexisting and acquired face representations: the N250 event-related potential as an index of face familiarity. J. Cogn. Neurosci. 18, 1488–1497. 10.1162/jocn.2006.18.9.1488 16989550

[B61] Tolba A. El-Baz A. El-Harby A. (2006). Face recognition: a literature review. Int. J. Signal Process. 2, 88–103.

[B62] Wang C. Guan C. Zhang H. (2005). P300 Brain-Computer Interface Design for Communication and Control Applications, in Proceedings of the 27th Annual International Conference of the IEEE Engineering in Medicine and Biology Society (EMBC) (Shanghai: IEEE), 5400–5403. 10.1109/IEMBS.2005.1615703 17281473

[B63] Wang Y. Jiang L. Wang Y. Cai B. Wang Y. Chen W. (2015). An iterative approach for EEG-based rapid face search: a refined retrieval by brain computer interfaces. IEEE Transactions Auton. Mental Develop. 7, 211–222. 10.1109/TAMD.2015.2446499

[B64] Wu Z. Ke Q. Sun J. Shum H. Y. (2010). Scalable face image retrieval with identity-based quantization and multi-reference re-ranking, in Proceedings of the IEEE Conference on Computer Vision and Pattern Recognition (CVPR) (San Francisco, CA: IEEE), 3469–3476. 10.1109/CVPR.2010.5539976

[B65] Zhang P. Wang X. Li X. Dai P. (2016). EEG feature selection based on weighted-normalized mutual information for mental fatigue classification, in Proceedings of the IEEE International Instrumentation and Measurement Technology Conference (I2MTC) (Taipei: IEEE), 1–6.

[B66] Zhang X. Gao Y. (2009). Face recognition across pose: a review. Pattern Recog. 42, 2876–2896. 10.1016/j.patcog.2009.04.017

[B67] Zhao W. Chellappa R. Phillips P. J. Rosenfeld A. (2003). Face recognition: a literature survey. ACM comput. Surv. 35, 399–458. 10.1145/954339.954342

[B68] Zheng X. Mondloch C. J. Segalowitz S. J. (2012). The timing of individual face recognition in the brain. Neuropsychologia 50, 1451–1461. 10.1016/j.neuropsychologia.2012.02.030 22410414

