# Supplementary material for: Spatial-Temporal Feature Analysis on Single-Trial Event Related Potential for Rapid Face Identification
Source: Front Comput Neurosci. 2017 Nov 27;11:106. doi: 10.3389/fncom.2017.00106 (PMC5711855; doi:10.3389/fncom.2017.00106)
Supplement: Supplementary file 1 [file Image1.PDF]

supplemental file

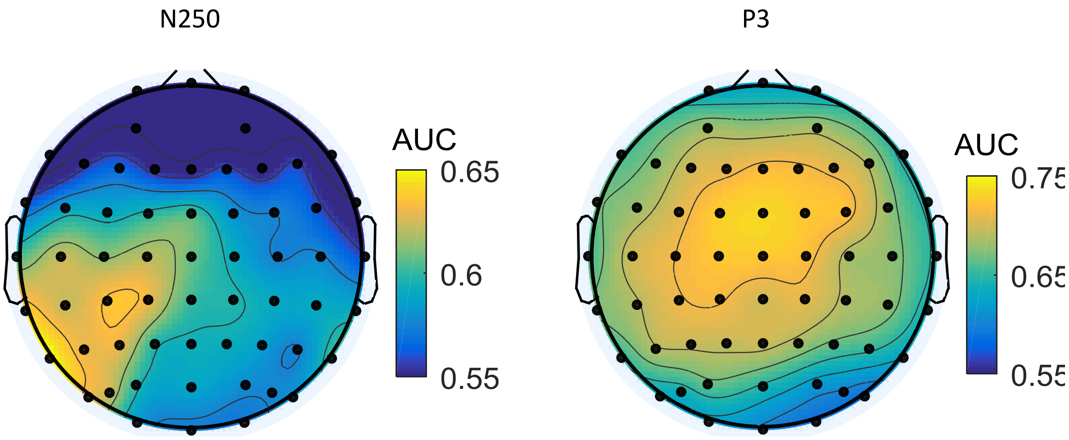

Fig. 1. The scalp topograph of performance of single-trial ERP detection using N250 or P3 component in every single electrode. The performace averaged across subjects is shown.
